# *Notes from the Field:* Occupational Hazards Associated with Harvesting and Processing Cannabis — Washington, 2015–2016

**DOI:** 10.15585/mmwr.mm6708a7

**Published:** 2018-03-02

**Authors:** Kerton R. Victory, James Couch, Brian Lowe, Brett J. Green

**Affiliations:** ^1^Office of the Director, Emergency Preparedness and Response Office, National Institute for Occupational Safety and Health, CDC; ^2^Division of Surveillance, Hazard Evaluations, and Field Studies, National Institute for Occupational Safety and Health, CDC; ^3^Division of Applied Research and Technology, National Institute for Occupational Safety and Health, CDC; ^4^Health Effects Laboratory Division, National Institute for Occupational Safety and Health, CDC.

Although the possession, use, and sale of all forms of cannabis are illegal under U.S. federal law, since 2012, multiple states have legalized the retail sale of cannabis for medical and recreational use (*1*). Previous research studies have indicated that Δ9-tetrahydrocannabinol (Δ9-THC), the principal psychoactive constituent of cannabis, can cause acute and chronic health effects (*2*). However, health effects from long-term occupational exposures to cannabis during harvesting and processing are unknown, in part because most studies have focused primarily on nonoccupational settings (*3*). In June 2015, the National Institute for Occupational Safety and Health (NIOSH) received a request for a Health Hazard Evaluation (HHE) from a representative of the United Food and Commercial Workers International Union to evaluate potential health and safety hazards associated with harvesting and processing cannabis at an outdoor farm.

In response to the request, NIOSH visited the farm in August and October 2015. The farm was located in Washington; the state had legalized cannabis for medicinal use in 1998 and recreational use in 2012. At the time of the HHE, the farm was operated by the owner and three employees. The 5-acre farm did not use any pesticides and grew cannabis, vegetables, and fruits. During the visit, the owner and all three employees were interviewed about their work, safety, and health concerns. Work practices were observed, and musculoskeletal risk factors for the hands, wrists, and shoulders during harvesting and processing tasks were evaluated. Digital force gauges and pinch gauges were used to assess manual hand forces during destemming, and a CyberGlove electrogoniometer glove (http://www.cyberglovesystems.com/) was used to assess dynamics and repetitive motion of the hand and fingers in trimming. Area and personal air samples were collected to test for gram-negative bacterial lipopolysaccharide (commonly referred to as endotoxin) and to determine bacterial and fungal diversity using 16S ribosomal RNA (16S rRNA) and fungal internal transcribed spacer (ITS) region gene sequencing, respectively (*4*). Exposure to these biologic hazards can increase the risk for allergic and respiratory symptoms (*2*). Surface wipe samples were collected and analyzed for Δ9-THC using ultra high performance liquid chromatography tandem mass spectrometry (*5*).

The owner and all three employees reported performing several tasks at the farm, including harvesting, bud stripping, and trimming. No one reported hand, wrist, or shoulder symptoms or other musculoskeletal problems. However, employees did express concerns about whether they might develop long-term musculoskeletal problems because of manually hand trimming cannabis. Harvesting tasks were observed and recorded by photograph and video. Analysis indicated that hand trimming of cannabis (Figure) involved low hand forces but was highly repetitive work.

**FIGURE:**
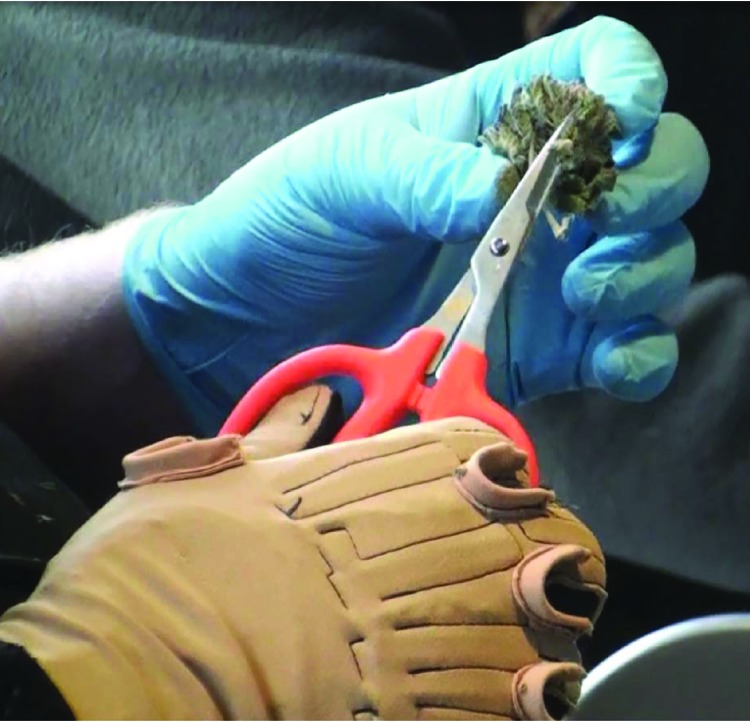
Hand trimming of cannabis flower using scissors while wearing a CyberGlove Photo/National Institute for Occupational Safety and Health

Personal, full-shift endotoxin air sample concentrations ranged from 2.8 to 37 endotoxin units per cubic meter, which was below the Dutch Expert Committee on Occupational Safety recommended occupational exposure limit of 90 endotoxin units per cubic meter. No U.S. occupational exposure limits for endotoxin are available. Analysis of bacterial diversity revealed outdoor area air samples composed of sequences derived from the phyla Proteobacteria (34%) and Actinobacteria (23%), whereas personal air samples were predominantly composed of sequences derived from the phylum Actinobacteria (47%). In contrast, sequencing of fungal ITS regions revealed a diversity composed of sequences predominantly assigned to the phylum Basidiomycota in outdoor (91%) and drying room samples (70%), whereas personal air samples had a lower fungal diversity predominantly composed of the Ascomycota fungal species, *Botrytis cinerea* (59%). This fungal species is a well-characterized aeroallergen and plant pathogen of cannabis. Δ9-THC was detected in all 27 surface sample wipes collected in cannabis production areas ranging from 0.17 to 210 *μ*g per 100 cm^2^.

The findings of this HHE indicated that the employees have exposures to highly repetitive work, most notably during hand trimming activities, which increase workers’ risk for musculoskeletal disorders (*6*). Worker exposure to aerosolized Actinobacteria and fungi such as *B. cinerea*, might also result from processing and hand trimming activities, which can increase the risk for allergic and respiratory symptoms, as has previously been observed in the cannabis processing industry (*2*). Δ9-THC surface wipe concentrations indicated the potential for dermal and ingestion exposures. However, the health implications from long-term occupational exposure to Δ9-THC are unknown. Detailed information is available in the final HHE report (https://www.cdc.gov/niosh/hhe/reports/pdfs/2015-0111-3271.pdf). The NIOSH HHE program (https://www.cdc.gov/niosh/hhe/) continues to evaluate potential hazards associated with the harvesting and processing of cannabis and will provide updated recommendations to educate employers and employees on the occupational hazards associated with the harvesting and processing of cannabis plants.
